# Supplementary material for: Molecular Evolution of Tubulins in Diatoms
Source: Int J Mol Sci. 2022 Jan 6;23(2):618. doi: 10.3390/ijms23020618 (PMC8776100; doi:10.3390/ijms23020618)
Supplement: Supplementary file 1 [file ijms-23-00618-s001.zip › ijms-1481722 supplementary final/S1_Table.pdf]

ID	Organism	Identity, %	Taxonomy current name
**ALPHA TUBULIN**			
	**GENOME DIATOM**		
FcTa	**Fracy1|169390**		*Fragilariopsis cylindrus* (Grunow ex Cleve) Helmcke & Krieger
PmTa1	**Psemu1|235379**	95,58	*Pseudo-nitzschia multiseries* (Hasle) Hasle
PmTa2	Psemu1|4917		*Pseudo-nitzschia multiseries*
PmtTa	**Psemulti_0108590**		*Pseudo-nitzschia multistriata* (H. Takano) H.Takano
PtTa	**Phatr2|54534**		*Phaeodactylum tricornutum* Bohlin
FrTa	***Fragilaria radians*|Synedra_Acus|21359**		*Fragilaria radians* (Kützing) D.M. Williams & Round
TpTa	**Thaps3|29304**		*Thalassiosira pseudonana* Hasle & Heimdal
	**TRANSCRIPTOME DIATOM**		
ApTa1	Amphiprora_paludosa_Strain_CCMP125|CAMPEP_0172440494		*Amphiprora paludosa* W. Smith Strain CCMP125
ApTa2	Amphiprora_paludosa_Strain_CCMP125|CAMPEP_0172442696		*Amphiprora paludosa* Strain CCMP125
ApTa3	**Amphiprora_paludosa_Strain_CCMP125|CAMPEP_0172471188**	79,69-93,53	*Amphiprora paludosa* Strain CCMP125
AsTa	**Amphiprora-sp|CAMPEP_0186488138**		*Amphiprora* sp. Ehrenberg
AcTa	**Amphora-coffeaeformis-CCMP127|CAMPEP_0186540212**		*Amphora coffeaeformis* (C. Agardh) Kützing CCMP127
AgTa1	**Asterionellopsis_glacialis_Strain_CCMP134|CAMPEP_0199876212**	55,88	*Asterionellopsis glacialis* (Castracane) Round Strain CCMP134
AgTa2	Asterionellopsis_glacialis_Strain_CCMP134|CAMPEP_0199886380		*Asterionellopsis glacialis* (Castracane) Round Strain CCMP134
AtsTa	**Attheya_septentrionalis_Strain_CCMP2084|CAMPEP_0198291474**		*Attheya septentrionalis* (Østrup) R.M.Crawford Strain CCMP2084
AusTa1a	**Aulacoseira_subarctica__Strain_CCAP_1002/5|CAMPEP_0172418740**	94,89	*Aulacoseira subarctica* (O. Müller) E.Y.Haworth Strain CCAP 1002/5
AusTa1b	Aulacoseira_subarctica__Strain_CCAP_1002/5|CAMPEP_0172421948		*Aulacoseira subarctica* Strain CCAP 1002/5
CbTa2	**Chaetoceros_debilis_Strain_MM31A-1|CAMPEP_0200885858**	73,17	*Chaetoceros debilis* Cleve Strain MM31A-1
CbTa1b	Chaetoceros_debilis_Strain_MM31A-1|CAMPEP_0200897568		*Chaetoceros debilis* Cleve Strain MM31A-1
CdiTa1	Chaetoceros_dichaeta_Strain_CCMP1751|CAMPEP_0198265246		*Chaetoceros dichaeta* Ehrenberg Strain CCMP1751
CdiTa2	**Chaetoceros_dichaeta_Strain_CCMP1751|CAMPEP_0198275888**	74,28	*Chaetoceros dichaeta* Strain CCMP1751

CnTa1b	**Chaetoceros_neogracile_Strain_CCMP1317|CAMPEP_0200987764**	72,95	*Chaetoceros neogracile* VanLandingham Strain CCMP1317
CnTa1a	Chaetoceros_neogracile_Strain_CCMP1317|CAMPEP_0201015148		*Chaetoceros neogracile* Strain CCMP1317
CafTa	**Chaetoceros-affinis-CCMP159|CAMPEP_0187038166**		*Chaetoceros affinis* Lauder Strain CCMP159
ChTa	**Corethron_hystrix_Strain_308|CAMPEP_0113305702**		*Corethron hystrix* Hensen Strain 308
CpTa	**Corethron_pennatum_Strain_L29A3|CAMPEP_0200323448**		*Corethron pennatum* (Grunow) Ostenfeld Strain L29A3
CauTa	**Craspedostauros_australis_Strain_CCMP3328|CAMPEP_0198134244**		*Craspedostauros australis* E.J. Cox Strain CCMP3328
CmTa	**Cyclotella_meneghiniana_Strain_CCMP_338|CAMPEP_0172265844**		*Cyclotella meneghiniana* Kützing Strain CCMP 338
DfTa	**Dactyliosolen_fragilissimus|CAMPEP_0184855548**		*Dactyliosolen fragilissimus* (Bergon) Hasle
DcTa	**Detonula_confervacea_Strain_CCMP_353|CAMPEP_0172309712**		*Detonula confervacea* (Cleve) Gran Strain CCMP 353
DbTa2	**Ditylum-brightwellii-GSO104|CAMPEP_0193942588**	73,11-91,78	*Ditylum brightwellii* (T. West) Grunow *GSO104*
DbTa1	Ditylum-brightwellii-GSO104|CAMPEP_0193943404		*Ditylum brightwellii* GSO104
DbTa1a	Ditylum-brightwellii-GSO104|CAMPEP_0193951558		*Ditylum brightwellii* GSO104
EaTa1	Eucampia_antarctica_Strain_CCMP1452|CAMPEP_019782462		*Eucampia antarctica* (Castracane) Mangin Strain CCMP1452
EaTa2	**Eucampia_antarctica_Strain_CCMP1452|CAMPEP_0197834012**	67,7	*Eucampia antarctica* Strain CCMP1452
EsTa1a	**Extubocellulus_spinifer_Strain_CCMP396|CAMPEP_0200463056**	97,32	*Extubocellulus spinifer* (Hargreaves & Guillard) Hasle, Stosch & Syvertsen Strain CCMP396
EsTa1b	Extubocellulus_spinifer_Strain_CCMP396|CAMPEP_0200507072		*Extubocellulus spinifer* Strain CCMP396
FksTa	**Fragilariopsis_kerguelensis_Strain_L2-C3|CAMPEP_0199350668**		*Fragilariopsis kerguelensis* (O'Meara) Hustedt Strain L2-C3
FkTa1	**Fragilariopsis-kerguelensis-L26_C5|CAMPEP_0188077452**	93,08	*Fragilariopsis kerguelensis* L26 C5
FkTa2	Fragilariopsis-kerguelensis-L26_C5|CAMPEP_0188155028		*Fragilariopsis kerguelensis* L26 C5
LdsTa	**Leptocylindrus_danicus_Strain_CCMP1856|CAMPEP_0196809184**		*Leptocylindrus danicus* Cleve Strain CCMP1856
LdTa1a	**Leptocylindrus_danicus_var._danicus_Strain_B650|CAMPEP_0116004598**	90,87	*Leptocylindrus danicus* var. *danicus* Strain B650
LdTa1b	Leptocylindrus_danicus_var._danicus_Strain_B650|CAMPEP_0116030842		*Leptocylindrus danicus* var. *danicus* Strain B650
LpTa	**Licmophora_paradoxa_Strain_CCMP2313|CAMPEP_0202441610**		*Licmophora paradoxa* (Lyngbye) C. Agardh Strain CCMP2313
MpTa	**Minutocellus_polymorphus_Strain_NH13|CAMPEP_0181036982**		*Minutocellus polymorphus* (Hargraves & Guillard) Hasle, Stosch, & Syvertsen Strain NH13
NpTa	**Nitzschia_punctata_Strain_CCMP561|CAMPEP_0199338790**		*Nitzschia punctata* (W. Smith) Grunow Strain CCMP561
NsTa	**Nitzschia_sp.|CAMPEP_0113462244**		*Nitzschia* sp. Hassall
OsTa	**Odontella_Sinensis_Strain_Grunow_1884|CAMPEP_0183309884**		*Odontella sinensis* (Greville) Grunow Strain Grunow 1884

OdTa	**Odontella|CAMPEP_0113546310**		*Odontella* sp.
PaTa2	Proboscia_alata_Strain_PI-D3|CAMPEP_0200137246		*Proboscia alata* (Brightwell) Sundström Strain PI-D3
PaTa1	**Proboscia_alata_Strain_PI-D3|CAMPEP_0200181428**	88,47	*Proboscia alata* Strain PI-D3
ParTa	**Pseudo-nitzschia_arenysensis_Strain_B593|CAMPEP_0116120156**		*Pseudo-nitzschia arenysensis* Quijano-Scheggia, Garcés, Lundholm Strain B593
PauTa	**Pseudo-nitzschia_australis_Strain_10249_10_AB|CAMPEP_0199657678**		*Pseudo-nitzschia australis* Frenguelli Strain 10249 10 AB
PdTa2	Pseudo-nitzschia_delicatissima_Strain_B596|CAMPEP_0116097972		*Pseudo-nitzschia delicatissima* (Cleve) Heiden Strain B596
PdTa1	**Pseudo-nitzschia_delicatissima_Strain_B596|CAMPEP_0116105316**	86,19	*Pseudo-nitzschia delicatissima* Strain B596
PfTa2	**Pseudo-nitzschia_fraudulenta_Strain_WWA7|CAMPEP_0199772816**	76,67	*Pseudo-nitzschia fraudulenta* (Cleve) Hasle Strain WWA7
PfTa2	Pseudo-nitzschia_fraudulenta_SPaTatrain_WWA7|CAMPEP_0199773496		*Pseudo-nitzschia fraudulenta* Strain WWA7
PhTa	**Pseudo-nitzschia_heimii_Strain_UNC1101|CAMPEP_0197177854**		*Pseudo-nitzschia heimii* Manguin Strain UNC1101
PpTa	**Pseudo-nitzschia_pungens_Strain_cf._cingulata|CAMPEP_0172375588**		*Pseudo-nitzschia pungens* (Grunow ex Cleve) Hasle Strain cf. cingulata
ScTa	**Skeletonema_costatum_Strain_1716|CAMPEP_0113389594**		*Skeletonema costatum* (Greville) Cleve Strain 1716
SgTa	**Skeletonema_grethea_Strain_CCMP_1804|CAMPEP_0201709312**		*Skeletonema grethea* Strain CCMP 1804
SjTa	**Skeletonema_japonicum_Strain_CCMP2506|CAMPEP_0201723980**		*Skeletonema japonicum* Zingone & Sarno Strain CCMP2506
SdTa	**Skeletonema-dohrnii-SkelB|CAMPEP_0192161800**		*Skeletonema dohrnii* Sarno & Kooistra Strain SkelB
SmaTa	**Skeletonema-marinoi-SkelA|CAMPEP_0192255302**		*Skeletonema marinoi* Sarno & Zingone Strain SkelA
SmeTa	**Skeletonema-menzelii-CCMP793|CAMPEP_0192262610**		*Skeletonema menzelii* Guillard, Carpenter & Reimann Strain CCMP793
ScsTa	**Stauroneis_constricta_Strain_CCMP1120|CAMPEP_0119547256**		*Stauroneis constricta* Ehrenberg Strain CCMP1120 (=*Craspedostauros alatus* Majewska & M.P. Ashworth 2018 according to [98])
SrTa1	Synedropsis_recta_cf_Strain_CCMP1620|CAMPEP_0119003500		*Synedropsis recta* G.R. Hasle, Medlin & E.E. Syvertsen cf Strain CCMP1620
SrTa2	**Synedropsis_recta_cf_Strain_CCMP1620|CAMPEP_0119013092**	93,16	*Synedropsis recta* Strain CCMP1620
TnTa1	**Thalassionema_nitzschioides_Strain_L26-B|CAMPEP_0200197008**	85,81	*Thalassionema nitzschioides* (Grunow) Mereschkowsky Strain L26-B
TnTa2	Thalassionema_nitzschioides_Strain_L26-B|CAMPEP_0200199412		*Thalassionema nitzschioides* Strain L26-B
TaTa1	**Thalassiosira_antarctica_Strain_CCMP982|CAMPEP_0200113142**	93,79	*Thalassiosira antarctica* Comber Strain CCMP982
TaTa2	Thalassiosira_antarctica_Strain_CCMP982|CAMPEP_0200128994		*Thalassiosira antarctica* Strain CCMP982
TgTa	**Thalassiosira_gravida_Strain_GMp14c1|CAMPEP_0200711806**		*Thalassiosira gravida* Cleve Strain GMp14c1

TmTa	**Thalassiosira_miniscula_Strain_CCMP1093|CAMPEP_0201026216**		*Thalassiosira minuscula* Krasske Strain CCMP1093
TpuTa1	**Thalassiosira_punctigera_Strain_Tpunct2005C2|CAMPEP_0172529678**	89,58	*Thalassiosira punctigera* (Castracane) Hasle Strain Tpunct2005C2
TpuTa2	Thalassiosira_punctigera_Strain_Tpunct2005C2|CAMPEP_0172572836		*Thalassiosira punctigera* Strain Tpunct2005C2
ToTa	**Thalassiosira-oceanica-CCMP1005|CAMPEP_0192906026**		*Thalassiosira oceanica* Hasle Strain CCMP1005
TrTa	**Thalassiosira-rotula-GSO102|CAMPEP_0193004208**		*Thalassiosira rotula* Meunier Strain GSO102
TxaTa1	**Thalassiothrix_antarctica_Strain_L6-D1|CAMPEP_0200963378**	92,27	*Thalassiothrix antarctica* Schimper ex Karsten Strain L6-D1
TxaTa2	Thalassiothrix_antarctica_Strain_L6-D1|CAMPEP_0200980512		*Thalassiothrix antarctica* Strain L6-D1
TdTa1	Triceratium_dubium_Strain_CCMP147|CAMPEP_0197433116		*Triceratium dubium* Brightwell Strain CCMP147
TdTa2a	Triceratium_dubium_Strain_CCMP147|CAMPEP_0197465128		*Triceratium dubium* Strain CCMP147
TdTa2b	**Triceratium_dubium_Strain_CCMP147|CAMPEP_0197466340**	79,91-90,22	*Triceratium dubium* Strain CCMP147
	**GENOME out group:**		
HsTa1a	NP_001257328		*Homo sapiens* Linnaeus
HsTa1b	NP_006073.2	99.5	*Homo sapiens*
HsTa1c	NP_116093.1	98	*Homo sapiens*
HsTa4a	NP_005991.1		*Homo sapiens*
HsTa3d	NP_525125.1	99.7	*Homo sapiens*
HsTa3c	NP_005992.1		*Homo sapiens*
HsTa3e	NP_997195.2	98.6	*Homo sapiens*
HsTa8	NP_061816.1		*Homo sapiens*
HsTaL3	NP_079079.1		*Homo sapiens*
AtTa1	NP_176654.1		*Arabidopsis thaliana* (L.) Heynh
AtTa2	NP_171974.1	88.6	*Arabidopsis thaliana*
AtTa3	NP_197478.1	91.7	*Arabidopsis thaliana*
AtTa6	NP_193232.1	88.4	*Arabidopsis thaliana*
**BETA TUBULIN**			
	**GENOME DIATOM**		
FcTb1	**Fracy1|275160**	90,83	*Fragilariopsis cylindrus*
FcTb2	Fracy1|274017		*Fragilariopsis cylindrus*
PmTb	**Psemu1|198602**		*Pseudo-nitzschia multiseries*
PtTb	**Phatr2|21122**		*Phaeodactylum tricornutum*

FrTb	**Fragilaria radians ǀSynedra_Acusǀ1562**		*Fragilaria radians*
TpTb1a	Thaps3ǀ31569		*Thalassiosira pseudonana*
TpTb2	**Thaps3ǀ8069**	78,33-97,18	*Thalassiosira pseudonana*
TpTb1b	Thaps3ǀ210		*Thalassiosira pseudonana*
	**TRANSCRIPTOME DIATOM**		
ApTb	**Amphiprora_paludosa_Strain_CCMP125ǀCAMPEP_0172472678**		*Amphiprora paludosa* Strain CCMP125
AcTb	**Amphora-coffeaeformis-CCMP127ǀCAMPEP_0186542886**		*Amphora coffeaeformis* Strain CCMP127
AgTb	**Asterionellopsis_glacialis_Strain_CCMP134ǀCAMPEP_0199870976**		*Asterionellopsis glacialis* Strain CCMP134
ArTb	**Astrosyne_radiata_Strain_13vi08-1AǀCAMPEP_0116850756**		*Astrosyne radiata* Ashworth & Lobban Strain 13vi08-1A
AtsTb	**Attheya_septentrionalis_Strain_CCMP2084ǀCAMPEP_0198305504**		*Attheya septentrionalis* Strain CCMP2084
AusTb	**Aulacoseira_subarctica__Strain_CCAP_1002/5ǀCAMPEP_0172423308**		*Aulacoseira subarctica* Strain CCAP 1002/5
CdTb1	Chaetoceros_debilis_Strain_MM31A-1ǀCAMPEP_0200898360		*Chaetoceros debilis* Strain MM31A-1
CdTb2	**Chaetoceros_debilis_Strain_MM31A-1ǀCAMPEP_0200898934**	82,02	*Chaetoceros debilis* Strain MM31A-1
CdiTb	**Chaetoceros_dichaeta_Strain_CCMP1751ǀCAMPEP_0198252006**		*Chaetoceros dichaeta* Strain CCMP1751
CnTb	**Chaetoceros_neogracile_Strain_CCMP1317ǀCAMPEP_0201001636**		*Chaetoceros neogracile* Strain CCMP1317
CsTb	**Chaetoceros_sp._Strain_GSL56ǀCAMPEP_0176481438**		*Chaetoceros* sp. Ehrenberg Strain GSL56
CafTb	**Chaetoceros-affinis-CCMP159ǀCAMPEP_0187034516**		*Chaetoceros affinis* Strain CCMP159
CcTb	**Chaetoceros-curvisetusǀCAMPEP_0187068936**		*Chaetoceros curvisetus* Cleve
ChTb	**Corethron_hystrix_Strain_308ǀCAMPEP_0113310782**		*Corethron hystrix* Strain 308
CpTb1	**Corethron_pennatum_Strain_L29A3ǀCAMPEP_0200307738**	99,32	*Corethron pennatum* Strain L29A3
CpTb2	Corethron_pennatum_Strain_L29A3ǀCAMPEP_0200330498		*Corethron pennatum* Strain L29A3
CwTb	**Coscinodiscus_wailesii_Strain_CCMP2513ǀCAMPEP_0172483548**		*Coscinodiscus wailesii* Gran & Angst Strain CCMP2513
CmTb1	Cyclotella_meneghiniana_Strain_CCMP_338ǀCAMPEP_0172271610		*Cyclotella meneghiniana* Strain CCMP 338
CmTb2	**Cyclotella_meneghiniana_Strain_CCMP_338ǀCAMPEP_0172279094**	79,23	*Cyclotella meneghiniana* Strain CCMP 338
DfTb	**Dactyliosolen_fragilissimusǀCAMPEP_0184871524**		*Dactyliosolen fragilissimus*
DbTb	**Ditylum-brightwellii-GSO104ǀCAMPEP_0193949392**		*Ditylum brightwellii* GSO104
EmTb	**Entomoneis_sp._Strain_CCMP2396ǀCAMPEP_0198143082**		*Entomoneis* Ehrenberg sp. Strain CCMP2396
EaTb	**Eucampia_antarctica_Strain_CCMP1452ǀCAMPEP_0197831824**		*Eucampia antarctica* Strain CCMP1452
EsTb	**Extubocellulus_spinifer_Strain_CCMP396ǀCAMPEP_0200503696**		*Extubocellulus spinifer* Strain CCMP396
FksTb	**Fragilariopsis_kerguelensis_Strain_L2-C3ǀCAMPEP_0199371270**		*Fragilariopsis kerguelensis* Strain L2-C3
FkTb	**Fragilariopsis-kerguelensis-L26_C5ǀCAMPEP_0188152368**		*Fragilariopsis kerguelensis* L26 C5

GoTb	**Grammatophora_oceanica_Strain_CCMP_410|CAMPEP_0194047338**		*Grammatophora oceanica* Ehrenberg Strain CCMP 410
LdsTb	**Leptocylindrus_danicus_Strain_CCMP1856|CAMPEP_0196803170**		*Leptocylindrus danicus* Strain CCMP1856
LdaTb2	Leptocylindrus_danicus_var._apora_Strain_B651|CAMPEP_0116056322		*Leptocylindrus danicus* var. *apora* Strain B651
LdaTb1	**Leptocylindrus_danicus_var._apora_Strain_B651|CAMPEP_0116057550**	81,08	*Leptocylindrus danicus* var. *apora* Strain B651
LdTb1	**Leptocylindrus_danicus_var._danicus_Strain_B650|CAMPEP_0116014292**	82,21	*Leptocylindrus danicus* var. *danicus* Strain B650
LdTb2	Leptocylindrus_danicus_var._danicus_Strain_B650|CAMPEP_0116018536		*Leptocylindrus danicus* var. *danicus* Strain B650
LpTb	**Licmophora_paradoxa_Strain_CCMP2313|CAMPEP_0202476408**		*Licmophora paradoxa* Strain CCMP2313
MpTb	**Minutocellus_polymorphus_Strain_RCC2270|CAMPEP_0185825564**		*Minutocellus polymorphus* Strain NH13
NsTb	**Nitzschia_sp.|CAMPEP_0113488770**		*Nitzschia* sp.
OsTb1a	Odontella_Sinensis_Strain_Grunow_1884|CAMPEP_0183298264		*Odontella sinensis* Strain Grunow 1884
OsTb1b	**Odontella_Sinensis_Strain_Grunow_1884|CAMPEP_0183307214**	94,42	*Odontella sinensis* Strain Grunow 1884
OdTb1a	**Odontella|CAMPEP_0113540332**	90,79	*Odontella sp.*
OdTb1b	Odontella|CAMPEP_0113570490		*Odontella sp.*
PaTb2	**Proboscia_alata_Strain_PI-D3|CAMPEP_0200146880**	84,62	*Proboscia alata* Strain PI-D3
PaTb1	Proboscia_alata_Strain_PI-D3|CAMPEP_0200161076		*Proboscia alata* Strain PI-D3
PiTb	**Proboscia_inermis_Strain_CCAP1064/1|CAMPEP_0171295370**		*Proboscia inermis* (F. Castracane) R.W. Jordan & R. Ligowski Strain CCAP1064/1
PauTb	**Pseudo-nitzschia_australis_Strain_10249_10_AB|CAMPEP_0199673840**		*Pseudo-nitzschia australis* Strain 10249 10 AB
PdTb1	Pseudo-nitzschia_delicatissima_Strain_B596|CAMPEP_0116093924		*Pseudo-nitzschia delicatissima* Strain B596
PdTb2	**Pseudo-nitzschia_delicatissima_Strain_B596|CAMPEP_0116101620**	81,18	*Pseudo-nitzschia delicatissima* Strain B596
PfTb	**Pseudo-nitzschia_fraudulenta_Strain_WWA7|CAMPEP_0199762250**		*Pseudo-nitzschia fraudulenta* Strain WWA7
PhTb1	**Pseudo-nitzschia_heimii_Strain_UNC1101|CAMPEP_0197185050**	82,77	*Pseudo-nitzschia heimii* Strain UNC1101
PhTb2	Pseudo-nitzschia_heimii_Strain_UNC1101|CAMPEP_0197196530		*Pseudo-nitzschia heimii* Strain UNC1101
PpTb	**Pseudo-nitzschia_pungens_Strain_cf._cingulata|CAMPEP_0172373056**		*Pseudo-nitzschia pungens* Strain cf. cingulata
SgTb	**Skeletonema_grethea_Strain_CCMP_1804|CAMPEP_0201685470**		*Skeletonema grethea* Strain CCMP 1804
SmeTb	**Skeletonema-menzelii-CCMP793|CAMPEP_0192278154**		*Skeletonema menzelii* Strain CCMP793
ScTb2	**Staurosira_complex_sp._Strain_CCMP2646|CAMPEP_0202493888**	77,51	*Staurosira* Ehrenberg complex sp. Strain CCMP2646
ScTb1	**Staurosira_complex_sp._Strain**_CCMP2646|CAMPEP_0202493888		*Staurosira* complex sp. Strain CCMP2646
SrTb	**Synedropsis_recta_cf_Strain_CCMP1620|CAMPEP_0119015808**		*Synedropsis recta* Strain CCMP1620
TnTb	**Thalassionema_nitzschioides_Strain_L26-B|CAMPEP_0200198590**		*Thalassionema nitzschioides* Strain L26-B
TaTb	**Thalassiosira_antarctica_Strain_CCMP982|CAMPEP_0200113766**		*Thalassiosira antarctica* Strain CCMP982
TgTb2	**Thalassiosira_gravida_Strain_GMp14c1|CAMPEP_0200705720**	79,46	*Thalassiosira gravida* Strain GMp14c1

TgTb1	Thalassiosira_gravida_Strain_GMp14c1|CAMPEP_0200730680		*Thalassiosira gravida* Strain GMp14c1
TmTb	**Thalassiosira_miniscula_Strain_CCMP1093|CAMPEP_0201049656**		*Thalassiosira minuscula* Strain CCMP1093
ToTb	**Thalassiosira-oceanica-CCMP1005|CAMPEP_0192888186**		*Thalassiosira oceanica* Strain CCMP1005
TrcTb1	**Thalassiosira-rotula-CCMP3096|CAMPEP_0192946304**	79,91	*Thalassiosira rotula* Strain CCMP3096
TrcTb2	Thalassiosira-rotula-CCMP3096|CAMPEP_0192984804		*Thalassiosira rotula* Strain CCMP3096
TrTb	**Thalassiosira-rotula-GSO102|CAMPEP_0192989516**		*Thalassiosira rotula* Strain GSO102
TwTb	**Thalassiosira-weissflogii-CCMP1010|CAMPEP_0193033132**		*Thalassiosira weissflogii* (Grunow) G.A. Fryxell & Hasle Strain CCMP1010
TwcTb	**Thalassiosira-weissflogii-CCMP1336|CAMPEP_0193074566**		*Thalassiosira weissflogii* Strain CCMP1336
TxaTb1	**Thalassiothrix_antarctica_Strain_L6-D1|CAMPEP_0200980980**	95,96	*Thalassiothrix antarctica* Strain L6-D1
TxaTb2	Thalassiothrix_antarctica_Strain_L6-D1|CAMPEP_0200980982		*Thalassiothrix antarctica* Strain L6-D1
TdTb1a	Triceratium_dubium_Strain_CCMP147|CAMPEP_0197435880		*Triceratium dubium* Strain CCMP147
TdTb2	Triceratium_dubium_Strain_CCMP147|CAMPEP_0197443726		*Triceratium dubium* Strain CCMP147
TdTb1b	**Triceratium_dubium_Strain_CCMP147|CAMPEP_0197468100**	77,08-89,46	*Triceratium dubium* Strain CCMP147
	**GENOME out group:**		
HsTbB6	NP_001290453		*Homo sapiens*
HsTbB	NP_821133		*Homo sapiens*
HsTbB1	NP_110400		*Homo sapiens*
HsTbB2A	NP_001060.1		*Homo sapiens*
HsTbB3	NP_006077		*Homo sapiens*
HsTbB4a	NP_001276052		*Homo sapiens*
HsTb8B	NP_817124.1		*Homo sapiens*
HsTbB2B	NP_821080	99.5	*Homo sapiens*
HsTbB4B	NP_006079	98.4	*Homo sapiens*
AtTb1	NP_177706.1		*Arabidopsis thaliana*
AtTb2	NP_568959.1		*Arabidopsis thaliana*
AtTb4	NP_199247.1		*Arabidopsis thaliana*
AtTb5	NP_564101.1		*Arabidopsis thaliana*
AtTb6	NP_196786.1		*Arabidopsis thaliana*
AtTb7	NP_180515.1		*Arabidopsis thaliana*
AtTb8	NP_001190373.1		*Arabidopsis thaliana*

AtTb9	NP_193821.1		*Arabidopsis thaliana*
**GAMMA TUBULIN**			
	**GENOME DIATOM**		
FcTg	**Fracy1|193621**		*Fragilariopsis cylindrus*
PmTg	**Psemu1|296046**		*Pseudo-nitzschia multiseries*
PmtTg	**Psemulti_0095950**		*Pseudo-nitzschia multistriata*
PtTg1	**Phatr2|44225**		*Phaeodactylum tricornutum*
FrTg	**Fragilaria radians |19726**		*Fragilaria radians*
TpTg	**Thaps3|29237**		*Thalassiosira pseudonana*
	**TRANSCRIPTOME DIATOM**		
AsTg	**Amphiprora-sp|CAMPEP_0186484566**		*Amphiprora* sp. Ehrenberg
CafTg	**Chaetoceros-affinis-CCMP159|CAMPEP_0187035934**		*Chaetoceros affinis* Lauder Strain CCMP159
CdTg	**Chaetoceros_debilis_Strain_MM31A-1|CAMPEP_0200887882**		*Chaetoceros debilis* Strain MM31A-1
CnTg	**Chaetoceros_neogracile_Strain_CCMP1317|CAMPEP_0200991790**		*Chaetoceros neogracile* Strain CCMP1317
CsTg	**Chaetoceros_sp._Strain_GSL56|CAMPEP_0176501072**		*Chaetoceros* sp. Strain GSL56
DfTg	**Dactyliosolen_fragilissimus|CAMPEP_0184870234**		*Dactyliosolen fragilissimus*
EsTg	**Extubocellulus_spinifer_Strain_CCMP396|CAMPEP_0200485434**		*Extubocellulus spinifer* Strain CCMP396
FksTg	**Fragilariopsis_kerguelensis_Strain_L2-C3|CAMPEP_0199389192**		*Fragilariopsis kerguelensis* Strain L2-C3
LdsTg	**Leptocylindrus_danicus_Strain_CCMP1856|CAMPEP_0196807940**		*Leptocylindrus danicus* Strain CCMP1856
LdTg	**Leptocylindrus_danicus_var._danicus_Strain_B650|CAMPEP_0116005642**		*Leptocylindrus danicus var. danicus* Strain B650
PdTg	**Pseudo-nitzschia_delicatissima_Strain_B596|CAMPEP_0116108306**		*Pseudo-nitzschia delicatissima* Strain B596
PpTg	**Pseudo-nitzschia_pungens_Strain_cf._cingulata|CAMPEP_0172375488**		*Pseudo-nitzschia pungens* Strain cf. cingulata
SjTg	**Skeletonema_japonicum_Strain_CCMP2506|CAMPEP_0201739406**		*Skeletonema japonicum* Strain CCMP2506
TrgTg	**Thalassiosira-rotula-GSO102|CAMPEP_0193020118**		*Thalassiosira rotula* Strain GSO102
TrTg	**Thalassiosira-rotula-CCMP3096|CAMPEP_0192947326**		*Thalassiosira rotula* Strain CCMP3096
TwTg	**Thalassiosira-weissflogii-CCMP1336|CAMPEP_0193065150**		*Thalassiosira weissflogii* Strain CCMP1336
TxaTg	**Thalassiothrix_antarctica_Strain_L6-D1|CAMPEP_0200972590**		*Thalassiothrix antarctica* Strain L6-D1
	**GENOME out group:**		
AtTg1	NP_191724.1		*Arabidopsis thaliana*
AtTg2	NP_196181.1	98.1	*Arabidopsis thaliana*
HsTg1	NP_001061.2		*Homo sapiens*

HsTg2	NP_057521	97.7	*Homo sapiens*

Reference

1. Majewska, R.; Ashworth, M.P.; Lazo-Wasem, E.; Robinson, N.J.; Rojas, L.; Van de Vijver, B.; Pinou, T. *Craspedostauros alatus* sp. nov., a new diatom (Bacillariophyta) species found on museum sea turtle specimens. *Diatom Res.* **2018**, *33*, 229–240.
