# Supplementary material for: Molecular Evolution of Tubulins in Diatoms
Source: Int J Mol Sci. 2022 Jan 6;23(2):618. doi: 10.3390/ijms23020618 (PMC8776100; doi:10.3390/ijms23020618)
Supplement: Supplementary file 1 [file ijms-23-00618-s001.zip › ijms-1481722 supplementary final/S7_Table.pdf]

Table S7a. Conservation in diatoms of the *H. sapiens* α-tubulin post-translationally modified residues. Large taxa are highlighted in color: green – Coscinodiscophyceae; red – Mediophyceae; orange – Bacillariophyceae, Urneidophycidae; blue – Bacillariophyceae, Fragilariophycidae; purple – Bacillariophyceae, Bacillariophycidae.

Posttranslational modification sites											
**ID**	**Acetylation**	**Detyrosination**	**Phosphorylation**	**Ubiquitinylation**	**Palmitoylation**	**Polyglutamylation**		**Interacting residues providing filament rigidity**		**Polyglycylation**	
	**K40**	**C-terminal Y451**	**Y432**	**K304**	**C376**	**E443**	**E445**	**K60**	**H283**	**E446**	**E448**
HsTa1a	K	Y	Y	K	C	E	E	K	H	E	G
**Outgroup**											
TdTa2b	A	Y	Y	A	C	E	E	K	H	D	G
TdTa2a	K	E	Y	K	C	E	E	K	H	E	
PfTa1	K	Y	Y	K	C		E	K	H	E	G
**Group α1**											
CbTa2	K	Y	Y	K	C	E	E	K	H	E	D
DbTa1	K	Y	Y	K	C	D	E	K	H	D	D
LdTa1a	K	Y	Y	K	C	E	E	K	H	D	D
AusTa1a	K	Y	Y	K	C	E	E	K	H	E	D
TdTa1	K	T	Y	K	C	E	E	K	H	D	D
EaTa2	K	Y	Y	K	C	E	E	K	H	D	E
PaTa2	K	Y	Y	K	C		E	K	H	D	E
TpTa	K	Y	Y	K	C		E	K	H	D	D
CmTa	K	Y	Y	K	C	E	E	K	H	D	D
CnTa1a	K	Y	Y	K	C	E	E	K	H	E	D
CdiTa1b	K	Y	Y	K	C		E	K	H	E	E
CpTa	K	Y	Y	K	C	E	E	K	H	D	D
ChTa	K	Y	Y	K	C	E	E	K	H	D	D
OdTa	K	Y	Y	K	C	E	E	K	H	D	E
OsTa	K	Y	Y	K	C	E	E	K	H	D	E
TaTa1	K	Y	Y	K	C	E	E	K	H	D	G
TrTa	G	Y	Y	K	C	D	E	K	H	D	G

TpuTa	K	Y	Y	K	C	E	E	K	H	D	G
TaTa2	K	Y	Y	K	C	E	E	K	H	D	D
TmTa	K	Y	Y	K	C	E	E	K	H	D	D
ToTa	K	Y	Y	K	C	E	E	K	H	D	D
TpuTa2	K	Y	Y	K	C		E	K	H	D	D
TgTa	K	Y	Y	K	C	E	E	K	H	D	D
DcTa	d	Y	Y	K	C	E	E	K	H	D	D
SgTa	K	Y	Y	K	C	E	D	K	H	F	D
SjTa	K	Y	Y	K	C	E	E	K	H	F	D
SmeTa	K	Y	Y	K	C	E	D	K	H	D	D
SmaTa	K	Y	Y	K	C	E	E	K	H	D	D
SdTa	K	Y	Y	K	C	E	E	K	H	D	D
ScTa	K	Y	Y	K	C	E	E	K	H	D	D
**Group $\alpha2$**											
DbTa1a	G	Y	Y	K	C	D	G	D	H	E	E
AusTa1b	K	Y	Y	K	C	D	E	K	H	E	D
EsTa1a	G	Y	Y	K	C		E	K	H	E	E
EsTa1b	G	Y	Y	K	C	E	E	K	H	D	E
MpTa	G	Y	Y	K	C		E	K	H	D	D
**Group $\alpha3$ ($\alpha3.1$)**											
AtsTa	K	Y	Y	K	C	E	E	K	H	D	D
LdTa1b	R	Y	Y	K	C	E	D	K	H	F	E
LdsTa	R	Y	Y	K	C	E	E	K	H	F	E
**Group $\alpha3$ ($\alpha3.2$)**											
PaTa1	I	Y	Y	K	C	E	E	K	H	D	D
DfTa	K	Y	Y	K	C	A	D	K	H	E	G
DbTa2	P	Y	Y	K	C	E	I	K	H	D	E
EaTa1	K	Y	Y	K	C		Q	K	H	I	G
CdiTa2	K	Y	Y	K	C		G	K	H	E	D
AgTa1	I	Y	Y	K	C	D	G	Q	Y	E	D

CbTa1	I	**Y**	**Y**	**K**	**C**		L	**K**	**H**	**E**	D
CafTa	I	**Y**	**Y**	**K**	**C**		L	**K**	**H**	**E**	**E**
CnTa1b	I	**Y**	**Y**	**K**	**C**		G	**K**	**H**	D	D
**Group α4**											
AgTa2	N	**Y**	**Y**	**K**	C	E	D	**K**	**H**	D	D
LpTa	S	**Y**	**Y**	**K**	C		**E**	**K**	**H**	D	D
TnTa2	A	**Y**	**Y**	**K**	C	**D**	D	**K**	**H**	F	D
TnTa1	N	**Y**	**Y**	**K**	C		D	**K**	**H**	**E**	D
TxaTa1	G	**Y**	**Y**	**K**	C		G	**K**	**H**	Y	D
TxaTa2	I	**Y**	**Y**	**K**	C		D	**K**	**H**	Y	D
FrTa	D	**Y**	**Y**	**K**	C		D	G	**H**	D	**E**
SrTa2	D	**Y**	**Y**	**K**	C		G	**K**	**H**	**E**	D
SrTa1	V	**Y**	**Y**	**K**	C	G	G	**K**	**H**	**E**	D
**Group α5**											
PtTa	**K**	**Y**	**Y**	**K**	C		A	S	**H**	G	D
AcTa	**K**	**Y**	**Y**	**K**	C		V	**K**	**H**	A	D
AsTa	I	**Y**	**Y**	**K**	C	A	**E**	**K**	**H**	G	D
ApTa1	I	**Y**	**Y**	**K**	C	A	**E**	**K**	**H**	G	D
ApTa3	I	**Y**	**Y**	**K**	C	A		**K**	**H**	**E**	D
ApTa2	I	**Y**	**Y**	**K**	C	A		**K**	**H**	D	D
ScsTa	**K**	**Y**	**Y**	**K**	C	A	G	**K**	**H**	G	D
CauTa	**K**	**Y**	**Y**	**K**	C		G	**K**	**H**	G	D
NpTa	I	**Y**	**Y**	**K**	C		D	**K**	**H**	L	
FcTa	I	**Y**	**Y**	**K**	C		**E**	**K**	**H**	L	
FkTa2	S	**Y**	**Y**	**K**	C		**E**	**K**	**H**	A	
NsTa	**K**	**Y**	**Y**	**K**	C		**E**	**K**	**H**	L	
FkTa1		**Y**	**Y**	**K**	C		**E**	**K**	**H**	A	
FksTa		**Y**	**Y**	**K**	C	G	**E**	**K**	**H**	L	
PfTa2	E	**Y**	**Y**	**K**	C	E	A	Q	**H**	I	
PhTa	S	**Y**	**Y**	**K**	C		L	Q	**H**	I	

ParTa		**Y**	**Y**	**K**	C		G	M	**H**	**E**	
PmTa2	A	**Y**	**Y**	**K**	C		A	**K**	**H**	**E**	**E**
PmTa1	A	**Y**	**Y**	**K**	C		V	**K**	**H**	**E**	**E**
PmtTa	D	**Y**	**Y**	**K**	C		G	**K**	**H**	T	
PauTa	D	**Y**	**Y**	**K**	C	A	A	**K**	**H**	G	
PpTa	H	**Y**	**Y**	**K**	C		G	Q	**H**	I	Y
PdTa1	N	**Y**	**Y**	**K**	C		Y	**K**	**H**	**E**	**E**
PdTa2	N	**Y**	**Y**	**K**	C		Y	**K**	**H**	**E**	**E**

Table S7b. Conservation in diatoms of the *H. sapiens* β-tubulin post-translationally modified residues. Large taxa are highlighted in color: green – Coscinodiscophyceae; red – Mediophyceae; blue – Bacillariophyceae, Fragilariophycidae; purple – Bacillariophyceae, Bacillariophycidae.

Posttranslational modification sites								
	**Acetylation**	**Polyamination**	**Phosphorylation**	**Polyglutamylation**	**Polyglutamylation/ Polyglycylation**	**Polyglutamylation**	**Polyglycylation**	
ID	**K252**	**Q15**	**S172**	**E435**	**E438**	**E441**	**E437**	**E439**
HsTb1	**K**	**Q**	**S**	**E**	**E**	**E**	**E**	T
**Outgroup**								
TdTb2	**K**	**Q**	**S**	**E**	**D**	**D**	**E**	D
ScTb1	**K**	**Q**	**S**	**E**	**D**	**D**	D	**E**
**Group β1**								
PaTb2	**K**	**Q**	**S**	**E**	**D**	**E**	**E**	D
LdTb1	**K**	**Q**	**S**	**E**	**D**	**E**	**E**	D
LdaTb2	**K**	**Q**	**S**	**E**	**D**	**E**	**E**	D
SmeTb	**K**	**Q**	**S**					
SgTb	**K**	**Q**	**S**	**E**	**D**		**E**	D
CdiTb	**K**	**Q**	**S**	**E**	**D**	**D**	**E**	D
CdTb2	**K**	**Q**	**S**	**E**	**D**	**E**	**E**	D
TpTb2	**K**	**Q**	**S**	**E**	**D**	**E**	**E**	D
CmTb2	**K**	**Q**	**S**	**E**	**D**	**E**	**E**	D
CwTb	**K**	**Q**	**S**	**E**	**D**	**E**	**E**	D
TgTb2	**K**	**Q**	**S**	**E**	**D**	**E**	**E**	D
TrcTb2	**K**	**Q**	**S**	**E**	**D**	**E**	**E**	D
**Group β2**								
DbTb	**K**	**Q**	**S**	G	**E**	Y	D	D
MpTb	**K**	**Q**	**S**	**E**	**D**	**D**	**E**	
EsTb	**K**	**Q**	**S**	**E**	G	**E**	**E**	G
OsTb1a	**K**	**Q**	**S**	**E**	**E**	**E**	**E**	**E**
OsTb1b	**K**	**Q**	**S**	**E**	**E**	**E**	**E**	**E**
AusTb	**K**	**Q**	**S**	**E**	**E**	G	**E**	**E**

AtsTb	**K**	**Q**	**S**	**E**	**D**	F	**E**	D
PaTb1	**K**	**Q**	**S**	**E**	**E**	**E**	**E**	**E**
PiTb	**K**	**Q**	**S**	**E**	**E**	**E**	**E**	**E**
ChTb	**K**	**Q**	**S**	**E**	**E**	**E**	**E**	**E**
TdTb1b	**K**	**Q**	**S**	**D**	G	**D**	**E**	**E**
OdTb1b	**K**	**Q**	**S**	**D**	G	**D**	**E**	**E**
TdTb1a	**K**	**Q**	**S**	**D**	G	**E**	**E**	**E**
OdTb1a	**K**	**Q**	**S**	**D**	G	**E**	**E**	**E**
CpTb1	**K**	**Q**	**S**	**D**	**E**	N	D	**E**
CpTb2	**K**	**Q**	**S**	**D**	**E**	N	D	**E**
**Group β3**								
LdaTb1	**K**	**Q**	**S**	**D**	**D**	**E**	D	D
LdsTb	**K**	**Q**	**S**	**E**	**E**	**E**	**E**	**E**
LdTb	**K**	**Q**	**S**	**E**	Q	**E**	D	**E**
TwTb	**K**	**Q**	**S**	**E**	Q	**E**	**E**	Q
TwcTb	**K**	**Q**	**S**	**E**	**E**	**E**	**E**	Q
CmTb1	**K**	**Q**	**S**	**D**	Q	**E**	D	Q
TpTb1a	**K**	**Q**	**S**	**E**	A	**E**		
TpTb1b	**K**	**Q**	**S**	L	T	N		
TaTb	**K**	**Q**	**S**	**E**	Q	**E**	**E**	Q
TmTb	**K**	**Q**	**S**	**E**	Q	**E**	**E**	Q
ToTb	**K**	**Q**	**S**	**D**	Y	**E**	D	Y
TgTb1	**K**	**Q**	**S**	**E**	Q	**E**	**E**	Q
TrcTb1	**K**	**Q**	**S**	**E**	**E**	**E**	**E**	**E**
TrTb	**K**	**Q**	**S**		Q	**E**	**E**	Q
**Group β4**								
CafTb	**K**	**Q**	**S**	**E**	**E**	**D**	**E**	E
CnTb	**K**	**Q**	**S**	**E**	**E**	**E**	**E**	E
CsTb	**K**	**Q**	**S**	**E**	**E**	**E**	**E**	E
CcTb	**K**	**Q**	**S**	**E**	**E**	**E**	**E**	E

CdTb1	K	Q	S	E	E	E	E	E
EaTb	K	Q	S	E	E	E	E	E
DfTb	K	Q	S	A	E	E	E	D
ScTb2	K	Q	S	E	E	E	E	E
AgTb	K	Q	S	E	D	E	E	D
**Group β5**								
FrTb	K	Q	S	D	D	N	D	D
SrTb	K	Q	S	D	A	D	D	A
TnTb	K	Q	S	D	D	D	D	D
TxaTb1	K	Q	S	E	D	D	E	D
TxaTb2	K	Q	S	E	E	E	E	E
GoTb	K	Q	S	E	D	E	E	D
ArTb	K	Q	S	D	E	E	D	E
LpTb	K	Q	S	D	D	D	D	D
**Group β6**								
PtTb	K	Q	S	D	D	E	D	E
AcTb	K	Q	S	D	E	G	D	D
ApTb	K	Q	S	E	D	D	E	E
EmTb	K	Q	S	D		E	D	D
PdTb2	K	Q	S		D		F	E
PdTb1	K	Q	S	D	D		D	D
PhTb1	K	Q	S	D	D		D	D
PhTb2	K	Q	S	T	D		T	D
NsTb	K	Q	S	E	D	A	E	D
FcTb1	K	Q	S	D	D	E	D	D
FcTb2	K	Q	S	D	D	E	D	D
FkTb	K	Q	S	D	D	D	D	D
FksTb	K	Q	S	D	D	D	D	D
PfTb	K	Q	S	D	D	D	D	D
PauTb	K	Q	S	D	D	D	D	D

PmTb	**K**	**Q**	**S**	**D**	**D**	Q	D	D
PpTb	**K**	**Q**	**S**	**D**	**E**	**D**	D	**E**

Table S7c. Conservation in diatoms of the *H. sapiens* γ-tubulin post-translationally modified residues. Large taxa are highlighted in color: red – Mediophyceae; blue – Bacillariophyceae, Fragilariophycidae; purple – Bacillariophyceae, Bacillariophycidae.

SITE	S80	S131	T288	S361
HsTg1	**S**	**S**	**T**	**S**
**Group γ1**				
LdTg	G	**S**	**T**	A
LdsTg	G	**S**	**T**	A
**Group γ2**				
DfTg	G	**S**	**T**	A
CafTg	**S**	**S**	**T**	A
CnTg	**S**	**S**	**T**	A
CdTg	G	**S**	**T**	A
CsTg	G	**S**	**T**	A
**Group γ3**				
EsTg	**S**	**S**	**T**	A
TwTg	T	**S**	**T**	A
TpTg	G	**S**	**T**	A
SjTg	G	**S**	**T**	A
TrTg	G	**S**	**T**	A
TrgTg	G	**S**	**T**	A
**Group γ4**				
PtTg1		**S**	**T**	A
AsTg		**S**	**T**	A
TxaTg	G	**S**	**T**	A
FrTg	G	**S**	**T**	A
FcTg	G	**S**	**T**	A
FksTg	G	**S**	**T**	A
PdTg	G	**S**	**T**	A

PmTg	G	**S**	**T**	A
PmtTg	G	**S**	**T**	A
PpTg	G	**S**	**T**	A
