# Supplementary figures and images for: Molecular Evolution of Tubulins in Diatoms
Source: Int J Mol Sci. 2022 Jan 6;23(2):618. doi: 10.3390/ijms23020618 (PMC8776100; doi:10.3390/ijms23020618)

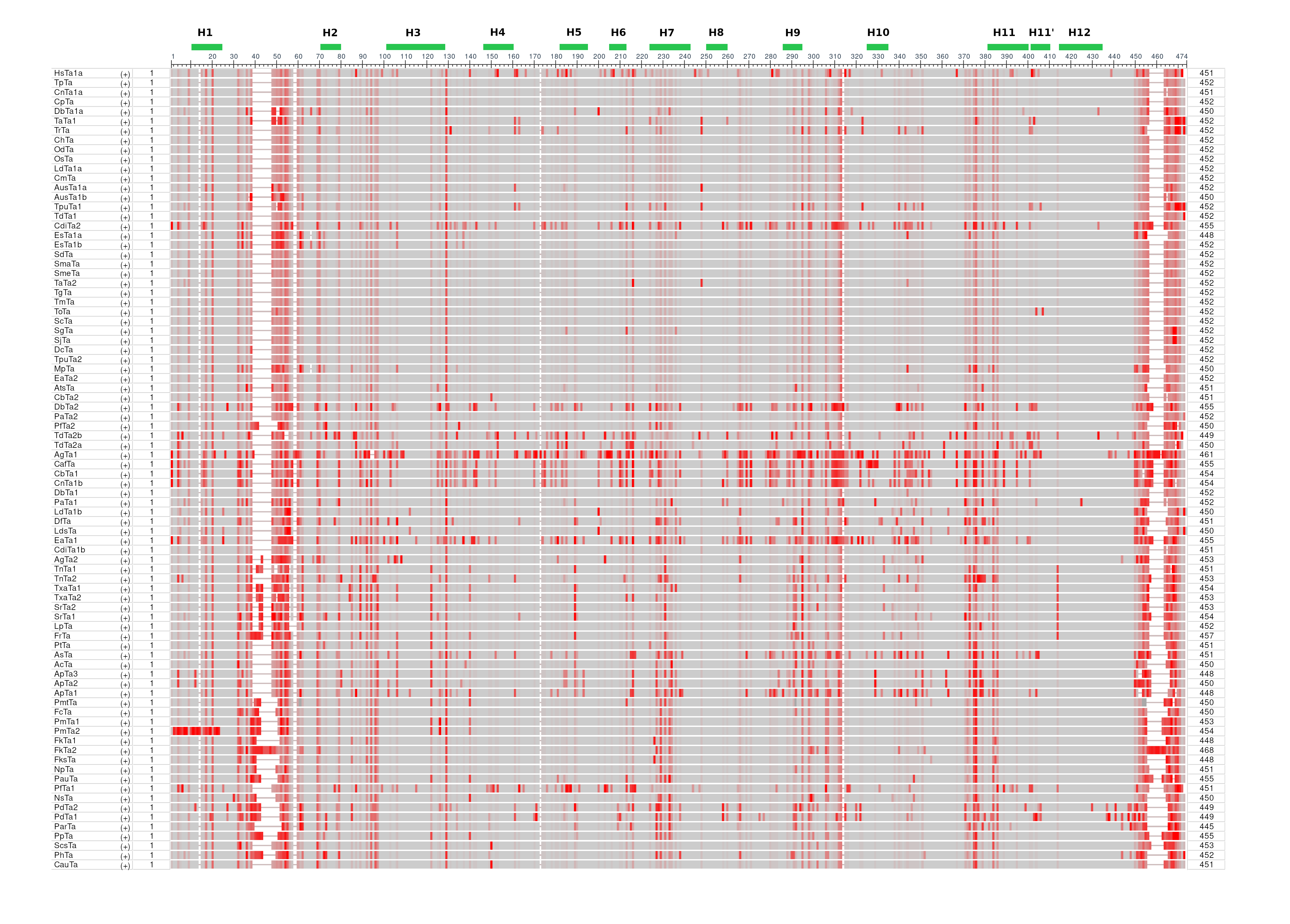

Supplement: Supplementary file 1 [file ijms-23-00618-s001.zip › ijms-1481722 supplementary final/S2A_Figure.tif]

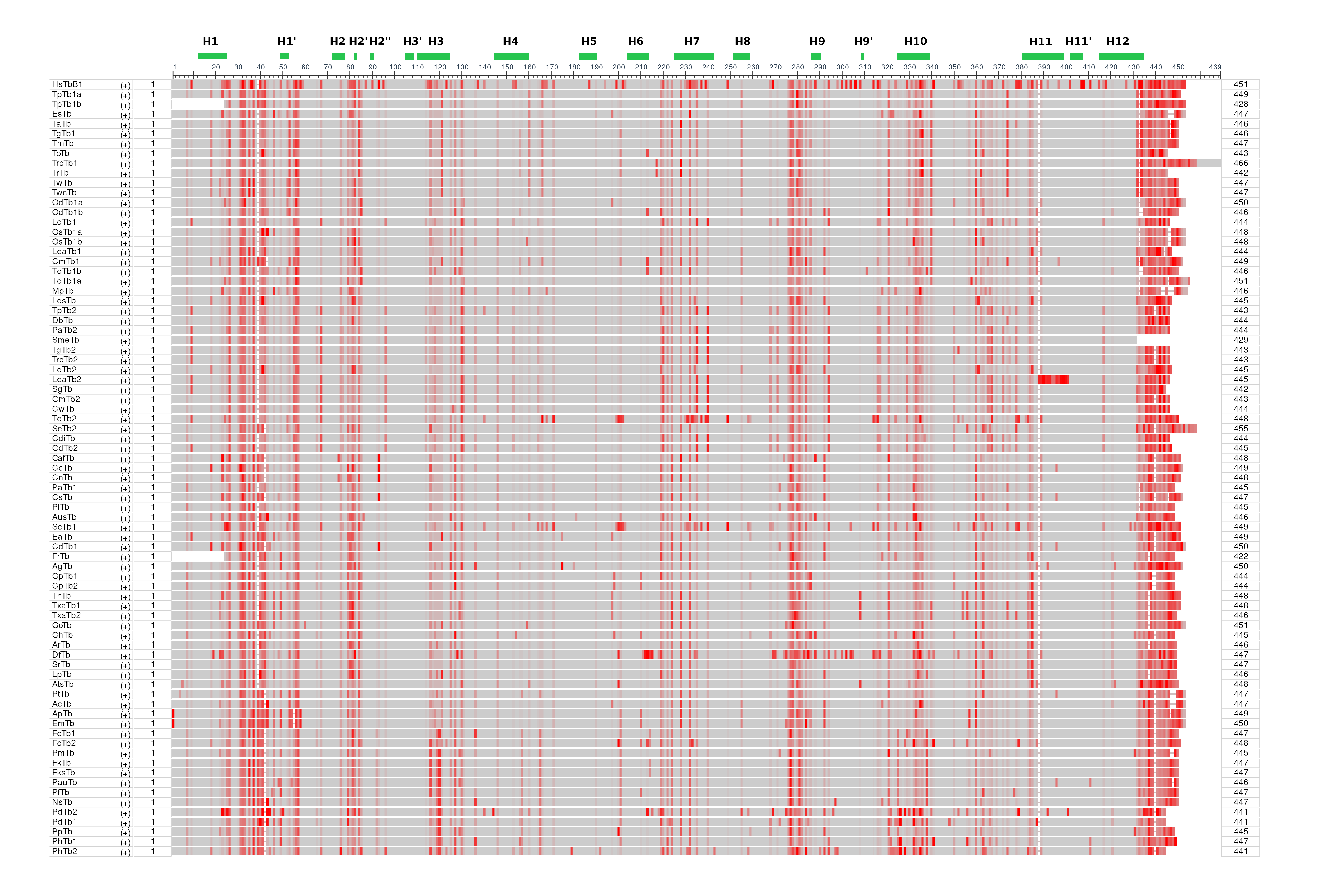

Supplement: Supplementary file 1 [file ijms-23-00618-s001.zip › ijms-1481722 supplementary final/S2B_Figure.tif]

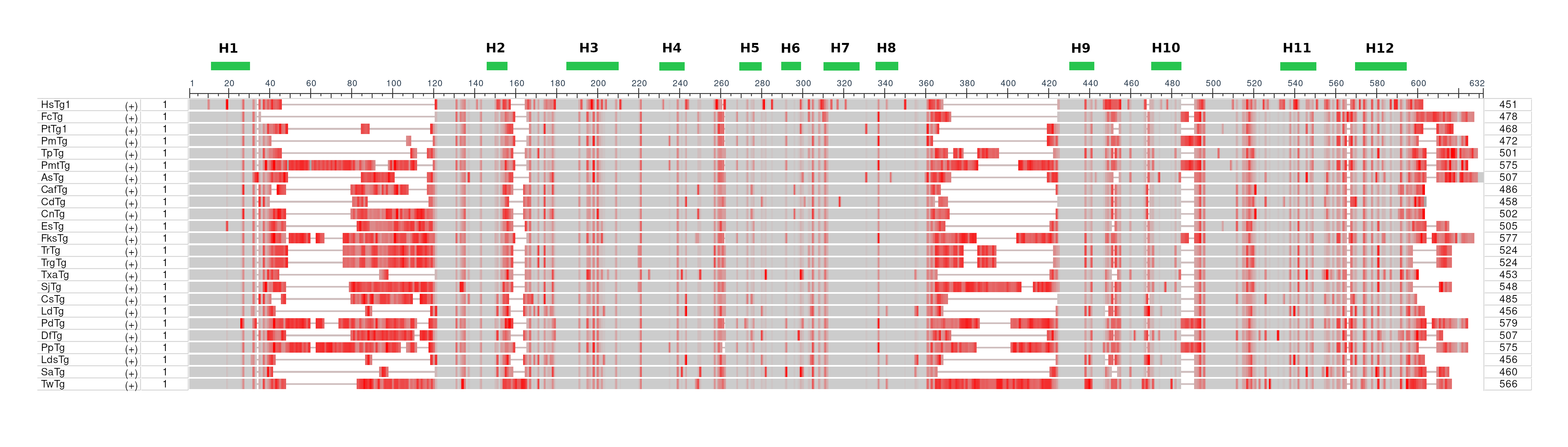

Supplement: Supplementary file 1 [file ijms-23-00618-s001.zip › ijms-1481722 supplementary final/S2C_Figure.tif]
